# The effect of AP-2δ on transcription of the Prestin gene in HEI-OC1 cells upon oxidative stress

**DOI:** 10.1186/s11658-019-0170-0

**Published:** 2019-06-26

**Authors:** Xuan Luo, Yun Xia, Xu-Dong Li, Jun-Yi Wang

**Affiliations:** 1 Department of Labor Health and Environmental Hygiene, School of Public Health, Guangdong Pharmaceutical University, Guangzhou, 510310 China; 2 Department of Labor Health and Environmental Hygiene, School of Public Health, Guangdong Pharmaceutical University, Guangzhou, 510310 China; 3 Key Laboratory, Occupational Disease Prevention and Control of Hospital of Guangdong Province, Guangzhou, 510300 China

**Keywords:** Prestin, AP-2δ, Oxidative stress, Transcriptional regulation, HEI-OC1 cells

## Abstract

**Background:**

The study aimed to investigate the effect of oxidative stress on Prestin expression, and explore the transcription factors (TFs) that are involved in regulating the expression of *Prestin* in House Ear Institute-Organ of Corti 1 (HEI-OC1) cells upon oxidative stress.

**Methods:**

Quantitative real-time polymerase chain reaction (qRT-PCR) and Western blot were used to detect the expression level of Prestin. Reverse chromatin immunoprecipitation (reverse ChIP) assay was performed to identify proteins that could bind to the Prestin gene. Small interfering RNA (siRNA) and chromatin immunoprecipitation (ChIP) experiments were used to further verify the results. HEI-OC1 cells were incubated with four different concentrations of tert-butyl hydroperoxide (t-BHP) for 24 h or 48 h to construct the oxidative stress model.

**Results:**

Oxidative stress induced Prestin increase at the mRNA level but with a concomitant decrease at the protein level. TF activating enhancer binding protein-2δ (AP-2δ) screened by reverse ChIP assay was demonstrated to bind to transcriptional start site 1441 of the *Prestin* promoter region and negatively regulate the expression of Prestin by siRNA and ChIP experiments. Furthermore, AP-2δ was down-regulated under oxidative stress.

**Conclusions:**

In conclusion, oxidative stress inhibits the expression of Prestin protein, and the transcription mechanism is triggered to compensate for the loss of Prestin protein. AP-2δ is one of the important TFs that suppresses transcription of the Prestin gene, and AP-2δ suppression further boosted Prestin mRNA activation under oxidative stress.

**Electronic supplementary material:**

The online version of this article (10.1186/s11658-019-0170-0) contains supplementary material, which is available to authorized users.

## Background

The World Health Organization reported that 5% of the world population, which is equal to 360 million people, had hearing issues in 2015. Hearing loss has a serious impact on quality of life and the economy of society [1]. Sensory deafness (including noise-induced deafness, drug-induced deafness, senile deafness, sudden deafness, etc.) is a category of hearing loss disability and accounts for a large proportion of deafness. The fundamental cause is the irreversible death of mammalian cochlea outer hair cells (OHCs) [2, 3]. The electromotility of OHCs converts electrical signals into mechanical energy and gives feedback to the basement membrane, further enhancing the vibrations of the basilar membrane and amplifying the sensitivity of the hair cells to mechanical stimulation. The hearing threshold can be increased by 40–50 dB (dB) through this local mechanical amplification, resulting in the exquisite hearing sensitivity and frequency selectivity of the mammalian cochlea [4, 5]. This effect is achieved through the exclusive motor protein of OHCs, Prestin [6].

In 2000, Zheng et al. [7] first isolated a gene encoding the motor protein of cochlea OHCs, *Prestin*. Prestin, an important sense-function protein that is specifically expressed in OHCs, is the OHC motor molecule [6, 8]. Seymour et al. [8] found that the electromotility of the cells expressing Prestin is related to the expression level of Prestin protein. Cell morphology changes with the protein conformation, contributing to the contact of OHC stereocilia and tectorial membrane and directly affecting the degree of amplification and the sensitivity of the audio signal [9, 10].

Noise, ionizing radiation, ototoxic drugs and other factors can cause sensory deafness and alterations of the expression of the cochlea OHC Prestin. The expression level of Prestin mRNA was elevated in cochlea OHCs of rats and guinea pigs exposed to impulsive noise [11], while the expression level of Prestin mRNA was lower in the OHCs of mice stimulated by strong broadband noise [12]. Yang et al. [13] found OHC Prestin protein increased in mice exposed to ionizing radiation. The expression of OHC Prestin at the mRNA and protein levels showed a reversible increase in mice injected with sodium salicylate for a long time [14]. However, Prestin protein level decreased in the OHCs of mice that received long-term administration of kanamycin [15]. Xia et al. [16] discovered that the expression of Prestin at protein and mRNA levels increased after normalizing the number of residual OHCs in mice exposed to short-term broadband noise, and hypothesized that Prestin up-regulation may represent a generalized response to compensate for a state of hearing loss. Combined, these results indicate that the same exposure factors can induce different alterations of Prestin expression level in cochlea OHCs, and the specific mechanism of this variation is still unclear.

So far, the study of Prestin has mainly focused on the molecular structure of the protein and the influence of different factors on the expression of Prestin, whereas little is known about the transcriptional regulation mechanism of the Prestin gene. Transcriptional regulation is the first step of gene expression regulation, and it is the most fundamental way to control gene expression and involves many related factors, such as hormones, trans-regulatory factors, and so on. Thyroid hormone (TH) was the first substance found to be involved in the regulation of Prestin expression [17]. Transcription factors (TFs) are cofactors needed for RNA polymerase during transcription initiation. TFs can participate in the regulation of transcription by directly or indirectly recognizing cis-acting elements. TF Gata-3 [18], TF Brn-3c (Pou4f3) (a member of the POU family), TF C/ebpb (CCAAT/enhancer binding protein beta), and the calcium-dependent TF CaRF [19] contribute to regulating the expression of Prestin, but all the transcription factor binding sites remained undefined.

A high level of reactive oxygen species (ROS) produced by oxidative stress injury is an important mechanism of cochlea hair cell injury and the basic pathological process of various types of sensory deafness [20, 21]. ROS can cause polyunsaturated fatty acid peroxidation, DNA degradation and protein damage, which lead to cell dysfunction or even death [20]. It has been demonstrated that oxidative stress injury can induce hair cell death and influence post-transcriptional regulation. ROS can regulate the activation and signal transduction of pathways related to oxidative damage in cochlea hair cells [21, 22], for example, the c-jun N-terminal kinase (JNK) signal pathway [23]. Referring to many studies in the literature, the transcriptional regulation of the Prestin gene in auditory cells with oxidative stress injury has not been reported. Therefore, further study is necessary to understand the molecular mechanisms of Prestin expression variations induced by oxidative stress, an important cause of sensory deafness.

## Materials and methods

### Cell culture

House Ear Institute-Organ of Corti 1 (HEI-OC1) cells were kindly provided by Dr. Federico Kalinec (Los Angeles, CA, USA). HEI-OC1 cells can be cultured in permissive conditions (P-HEI-OC1) and non-permissive conditions (NP-HEI-OC1) [24]. Though Prestin protein is expressed in plasma member of NP-HEI-OC1 cells, which is more similar to OHCs, their cell viability decreases and cell death increases with respect to P-HEI-OC1 cells [25, 26]. In addition, Prestin expression and membrane localization are unstable during the differentiation process in NP-HEI-OC1 cells [24, 26]. Furthermore, HEI-OC1 cells are highly sensitive to pharmacological drugs or antibiotics, and the phenotype and biological response will change easily [25], so all treatment with HEI-OC1 cells was conducted under permissive conditions. In brief, the cells were cultured under permissive conditions (33 °C,10% CO_2_) in high-glucose Dulbecco’s Eagle’s medium (HyClone, Utah, USA) containing 10% fetal bovine serum (HyClone, Utah, USA) without antibiotics [24]. HEI-OC1 cells at 1 × 10^6^ cells/well were cultured in six-well plates and treated with different concentrations (0 μM, 50 μM, 100 μM, and 200 μM) of tert-butyl hydroperoxide (t-BHP) (Wako, Japan) for 24 h or 48 h. The cells were used at 70–80% confluence for the following experiments.

### Reverse chromatin immunoprecipitation and liquid chromatography mass spectrometry

1 × 10^9^ cells were cross-linked with 3% formaldehyde for 30 min at 37 °C to prepare chromatin. Chromatin supernatant was collected after ultrasonication and prewashing. The probes for Prestin were designed by EXIQON software online and labeled with desthiobiotin; they were provided by Biosense Biotech Company (Guangzhou, China). The sequences of probes were as follows: (a) 5-TACAGGCAGTCAGGTCATTAgt-3, (b) 5-TtgGTTCATCAGAAATGCTTcT-3, and (c) 5-gCACAGCAATCCACTTTACTAa-3 (the schematic diagram of the mouse Prestin gene shown in Additional file 1: Figure S1; it demonstrates the targeted positions of probes sequences). The probes were subjected to lock nucleic acid (LNA) modification treatment to increase the specificity. The LNA probes with a final concentration of 1 μM were added into the supernatant followed by a hybridization procedure (25 °C for 3 min, 70 °C for 6 min, 38 °C for 60 min, 60 °C for 2 min, 38 °C for 60 min, 60 °C for 2 min, 38 °C for 120 min, and 25 °C for 3 min). The supernatant was incubated for 12 h at 37 °C with avidin-conjugated magnetic beads. Subsequently, elution buffer was added to resuspend beads and protein was eluted by incubation with shaking. Protein samples were obtained after treating the samples in a water bath at 99 °C for 25 min with crosslinking reversal solution. Next, PAGE gel electrophoresis and Coomassie blue staining were performed to elute and detect the protein. The liquid chromatography mass spectrometry (LC-MS) steps followed a previously described method [27]. In brief, the gels were digested with trypsin, reduced with DTT and centrifuged. The peptide fragments were dissolved prior to LC-MS analysis and loaded into the mass spectrometer (Thermo Scientific, Wagtham, USA) directly to detect online; the general conditions were: resolution 70,000; AGC target 3e6; maximum IT 40 ms; scan range 350 to 1,800 m/z; MS2: resolution 17,500; AGC target 1e5; maximum IT 60 ms; TopN 20; and NCE/stepped NCE 27. The data acquired were transferred into MGF-formatted files and used to search the uniprot *Mus musculus* database with MASCOT.

### Quantitative real-time polymerase chain reaction

Total RNA was isolated and prepared using TRE-Trizol (Invitrogen, California, USA). Reverse transcription (RT) was performed after the concentration of total RNA was tested and met the requirements. cDNA was synthesized using PrimeScript II 1st Strand cDNA Synthesis Kit (TaKaRa, Japan) according to the manufacturer’s protocol followed by quantitative real-time polymerase chain reaction (qRT-PCR) using SYBR Premix Ex Taq II (TaKaRa, Japan). The primers for qRT-PCR (Sangon, Shanghai, China) are listed in Table 1. *GAPDH* was used as a housekeeping gene [13]. The 2^-ΔΔCt^ method was applied for relative quantitative gene expression [27].

**Table 1:** Primer sequences for qRT-PCR of target genes (F forward, R reverse, bp base pair)

Gene	Protein	Reference gene ID	Primer sequence (5 → 3)
*GAPDH*	GAPDH	NM_008084.2	F: TGTGTCCGTCGTGGATCTGA
			R: TTGCTGTTGAAGTCGCAGGAG
*Tfap2a*	AP-2α	21,418	F: ACTCGGTGGTACAAGTTCGG
			R: CGTGACGGTCCATAGCTGAA
*Tfap2b*	AP-2β	21,419	F: TTATGAGGCGGTGTAGGCAA
			R: AGACCTGCTCATCCGTCTCT
*Tfap2c*	AP-2γ	21,420	F: ACATGGGAGGAGGGTTGTTG
			R: TCCTGAGGGGACGAATCCTT
*Tfap2d*	AP-2δ	226,896	F: TCTGATCCGGGCAAAACCAT
			R: GTCGTGACGTATCTCCGCAT
*Tfap2e*	AP-2ε	332,937	F: TGGCTCGGGACTTTGGTTAC
			R: TCCTGAGCCATCAAGTCTGC
*Ebf3*	COE3	13,593	F: GTCAGAAGCCACTCCGTGTA
			R: TCAGCTCACTCCACACCAAC
*Tbx22*	TBX22	245,572	F: AGGGATGGAAGGATTCAGAGG
			R: TTGTGCTCACTTACATGGCCC
*Tbx5*	TBX5	21,388	F: CTCTAAGCCGTTCTGGAGCC
			R: GCGAGGTTCTATTCTCGCTC
*Prestin*	Prestin	80,979	F: CCTCTTGTTCCAGGGCCAAA
			R: TTGGGAGCACTGCAATCCAT

### Western blotting

The expression levels of Prestin and AP-2δ protein in cells were detected by western blot. Protein samples were prepared in lysis buffer (50 mM Tris (pH 7.4), 150 mM NaCl, 1% Triton X-100, 1% sodium deoxycholate,0.1% SDS, 1 mM phenylmethylsulfonyl fluoride, PMSF), dissociated on ice for 30 min and centrifuged at 12,000 rpm for 10 min at 4 °C. A total of 40–60 μg of supernatant was mixed with 5X loading buffer and electrophoresed on 10% SDS-PAGE then transferred to polyvinylidene fluoride (PVDF) membranes (Merk Millipore, USA). The membrane, blocked with 5% non-fat milk, was incubated with goat anti-Prestin (1:500; Santa Cruz, USA), goat anti-AP-2δ (1:1000; Santa Cruz, USA) and anti-GAPDH (11,000; CWBIO, China) at 4 °C overnight. Then, the corresponding secondary antibodies with HRP (15000) conjugates were added and incubated for 1 h at 37 °C. Finally, the signal was detected with BeyoECLPlus (Beyotime, China), analyzed by ImageJ software, and normalized for GAPDH staining.

### Chromatin immunoprecipitation

HEI-OC1 cells were cross-linked with 1% formaldehyde for 10 min at 37 °C, the chromatin was prepared as described previously [28] and sheared into 200–600 bp fragments using a Bioruptor (Diagenod, Belgium). The samples of 100 μl each in a tube were diluted 10-fold in chromatin immunoprecipitation (ChIP) dilution buffer and incubated with 1 μg of goat anti-AP-2δ (1:1000; Santa Cruz, USA) or 1 μg of control nonimmune IgG at 4 °C overnight. Subsequently, the DNA-protein complexes were precipitated and purified as described by Heimann et al. [29]: 2 μl of IP DNA or input DNA were templated for SYBR PCR reactions using the primers flanking the identified Prestin transcriptional start site (TSS) -2000 − + 500 bp. The primer sequences (forward and reverse, respectively) were as follows: S-1441 ChIP-Prestin, 5-CTTGTGGGGTGAGGGTAGAA-3, 5-GGAGAAACTGGCTGTCTTGC-3; S-784 ChIP-Prestin, 5-TTGTGGATGCTGGCATTAGC-3, 5-TAAGCTTGAGCAGCAGGTG-3.

### Small interfering RNA treatment

Combined with the results from reverse ChIP and qRT-PCR for the screened TFs, the TF activating enhancer binding protein-2δ (AP-2δ) was initially identified to participate in the regulation of *Prestin*. Three AP-2δ small interfering RNA (siRNA) fragments targeted to AP-2δ mRNA sequences were designed. The sequences of siRNA (forward and reverse, respectively) were as follows: siTfap2d-a, 5-UCAGUGAGAUGCUUAACUAUU-3, 5-UAGUUAAGCAUCUCACUGAUU-3; siTfap2d-b, 5-CAAACAGAAUCUAUUUCCAUU-3, 5-UGGAAAUAGAUUCUGUUUGUU-3; siTfap2d-c, 5-CUCAGUUCUACUUCCAAAUUU-3, 5-AUUUGGAAGUAGAACUGAGUU-3. A scrambled siRNA (5-GACGATGATTCGTATGTAAdTdT-3, 5-AATCATACGAATCATCGTCdTdT-3) served as a control group, and 2 × 10^5^ HEI-OC1 cells were transfected with the siRNA or control constructs and incubated in a six-well plate under permissive conditions without any treatment. After 24 h transfection, cells were collected and subjected to qRT-PCR and western blot experiments to measure the expression level of AP-2δ and to identify the siTfap2d fragment with the highest specificity. Afterward, the best specificity siTfap2d fragment validated by qRT-PCR and western blot as described above was used for evaluating the effect of AP-2δ on *Prestin*.

### Statistical analysis

Statistical analyses were performed with SPSS 21.0 software (IBM, USA). The results of Prestin and TF expression levels from three independent experiments were presented as the means±SD. All data were analyzed using Student’s *t*-test or one-way ANOVA followed by Tukey’s test to compare differences. A *P*-value < 0.05 was considered statistically significant.

## Results

### Prestin mRNA was up-regulated and Prestin protein was down-regulated in HEI-OC1 cells injured by oxidative stress

**Fig. 1:**
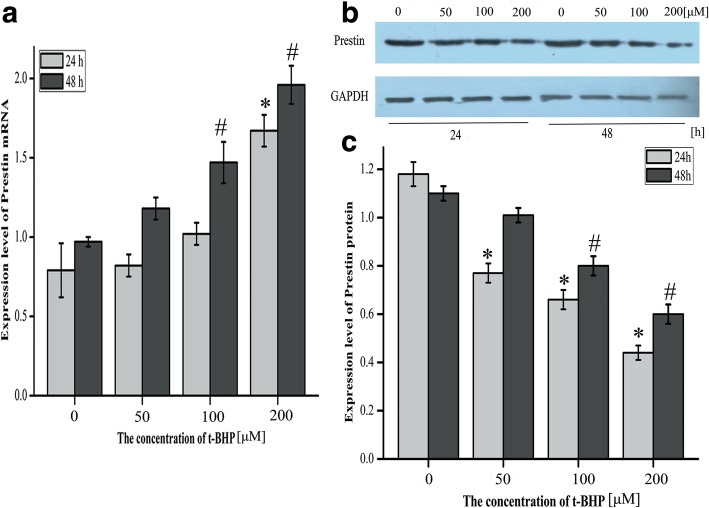
Expression level of Prestin in HEI-OC1 cells treated with t-BHP for 24 h and 48 h. **a** Expression level of Prestin mRNA in HEI-OC1 cells exposed to different concentrations of t-BHP (0 μM, 50 μM, 100 μM, 200 μM) for 24 h and 48 h. **b** Representative western blot of Prestin from cells treated with t-BHP. GAPDH was used as an endogenous control. These images came from the same gel. **c** Expression level of Prestin protein in HEI-OC1 cells exposed to t-BHP. The data were normalized to GAPDH expression, and they were presented as the means±SD; *n* = 3 each group. * and # represent *P* < 0.05 compared with the control group

To determine the effect of oxidative stress on the expression of Prestin, HEI-OC1 cells were exposed to different concentrations of t-BHP for 24 h or 48 h. The expression level of Prestin is shown in Fig. 1. At mRNA level, the quantity

of Prestin was increased in HEI-OC1 cells exposed to t-BHP in a dose-dependent manner (this meant that the higher the concentration of t-BHP was, the higher was the level of mRNA) (Fig. 1a). At protein level, oxidative stress induced a decrease of Prestin, and the expression level declined with the rise of t-BHP concentration (Fig. 1b). Furthermore, the Prestin mRNA expression level and protein level of the experimental groups in cultures after 48 h were higher than after 24 h (Student’s *t*-test, *P* < 0.05) .

### mRNA expression level of TFs probably modulated the Prestin gene in the state of oxidative stress

To identify proteins bound to the Prestin gene, reverse ChIP was performed in HEI-OC1 cells. 183 types of proteins (Additional file 1: Table S1) were recognized from the digested peptides using LC-MS. Among the recognized proteins only those 8 TFs (Table 1) with transcriptional function were chosen for further verification. Five of them belong to the activating enhancer binding protein 2 (AP2) family, namely, AP-2α, AP-2β, AP-2γ, AP-2δ and AP-2ε. The other three proteins were COE3 (transcription factor COE3), TBX5 (T-box transcription factor TBX5) and TBX22 (T-box transcription factor TBX22).

**Fig. 2:**
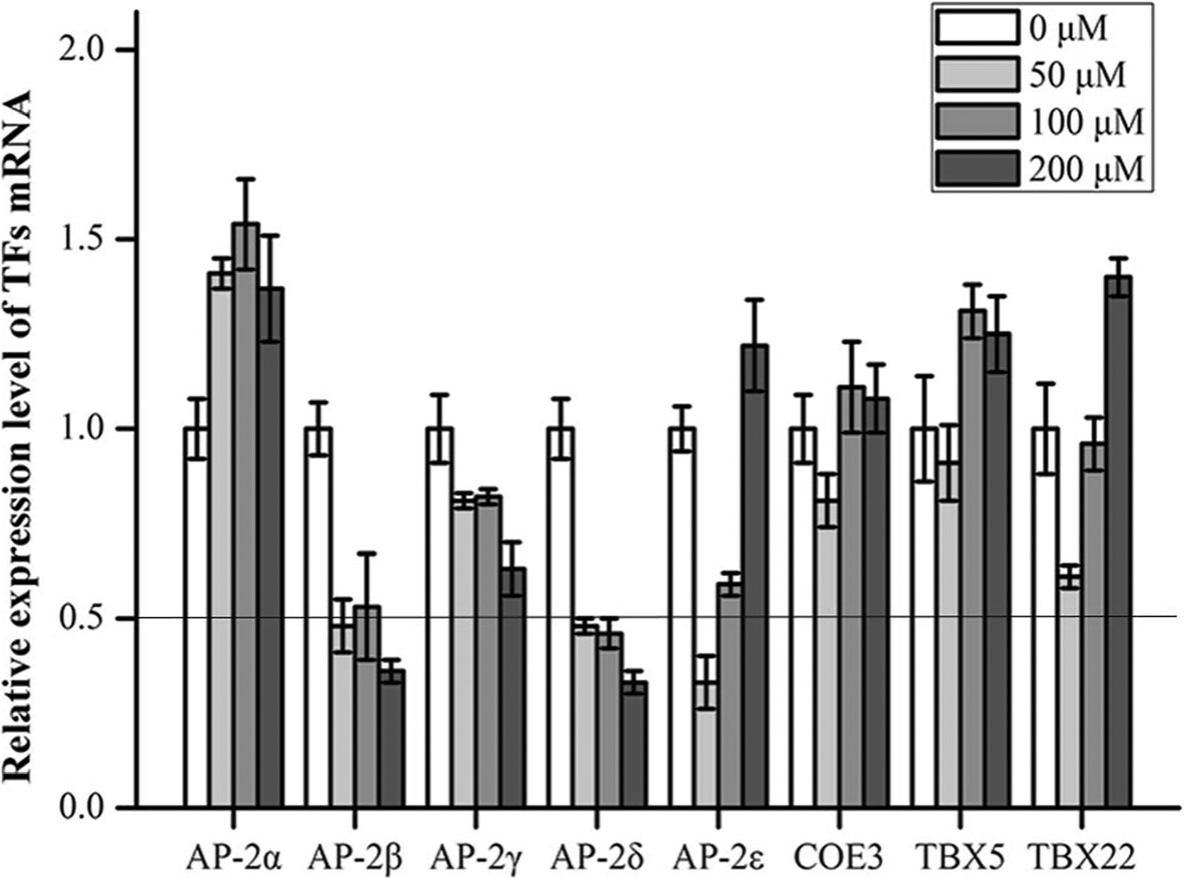
Relative expression level of TFs mRNA in HEI-OC1 cells treated with 0 μM, 50 μM, 100 μM, and 200 μM t-BHP for 24 h. mRNA level was assayed by qRT-PCR and normalized against the control group (the group treated with 0 μM t-BHP). The data are presented as the mean ± SD, *n* = 3 each group

The relative expression level of TF in experimental groups treated with t-BHP characterized by ≥2-fold up- or down-regulation was considered to be evidence that a given TF may modulate *Prestin*, and it was further processed for verification. Among the eight TFs, only AP-2δ met the requirement upon oxidative stress (Fig. 2). Additionally, the expression of AP-2δ mRNA in cells treated with t-BHP apparently decreased.

### ChIP assay confirmed AP-2δ as an interacting partner of the Prestin gene

**Fig. 3:**
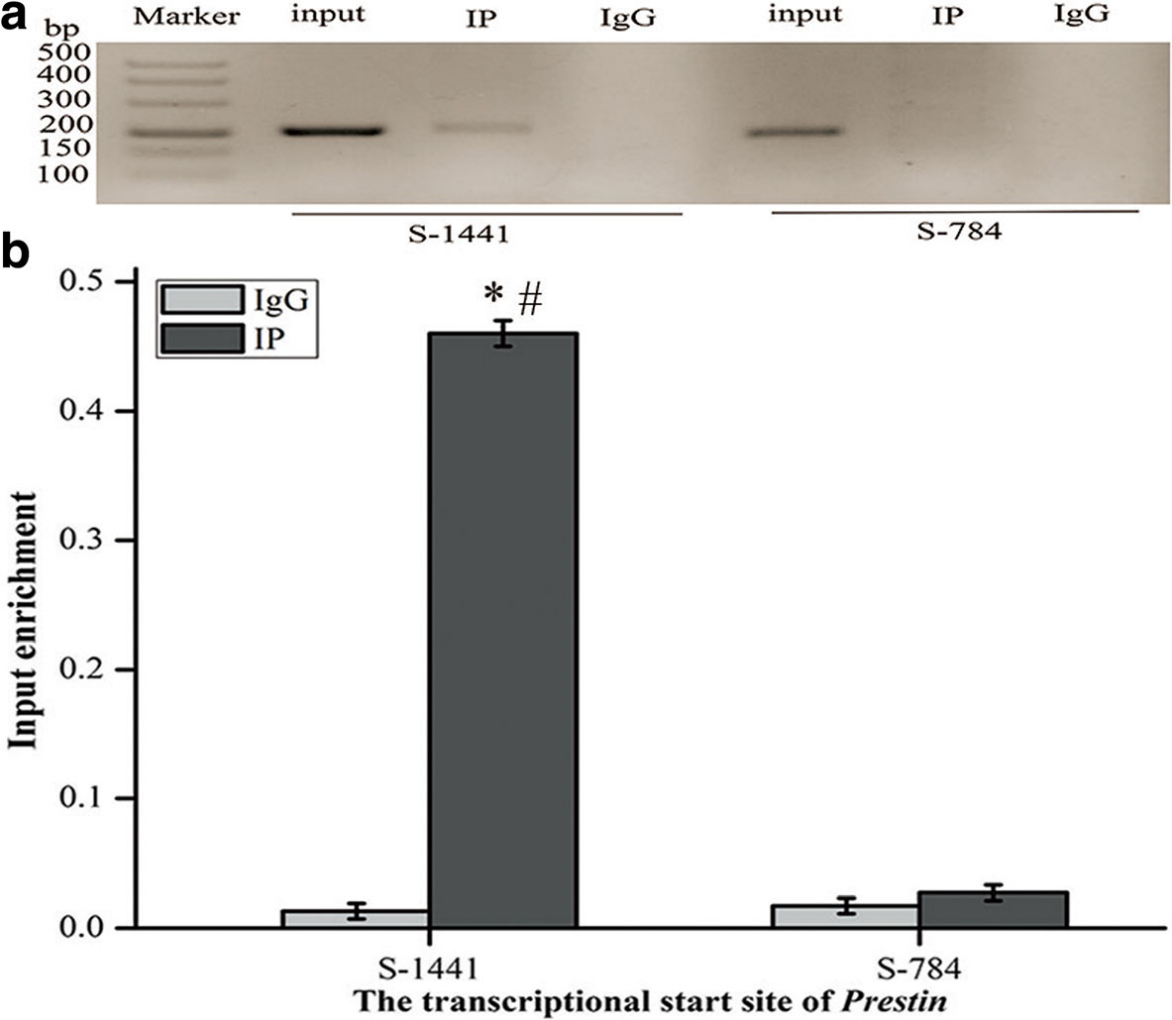
AP-2δ bound to *Prestin* in HEI-OC1 cells, as validated by ChIP assay. **a** The product of two primers flanking the transcriptional start site (TSS) of *Prestin* (S-1441 and S-784) was subjected to agarose gel electrophoresis after PCR. An input group, IP group, and IgG group were set up for each site. **b** Enrichment analysis of AP-2δ ChIP on *Prestin* regulatory regions. GAPDH was used as an endogenous control. Enrichment of AP-2δ-bound *Prestin* amount was normalized to corresponding amount in input group. The data were presented as the mean ± SD, *n* = 3 each group. * and # represent *P* < 0.05 compared with the IgG group of S-1441 and the IP group of S-784 respectively

**Fig. 4:**
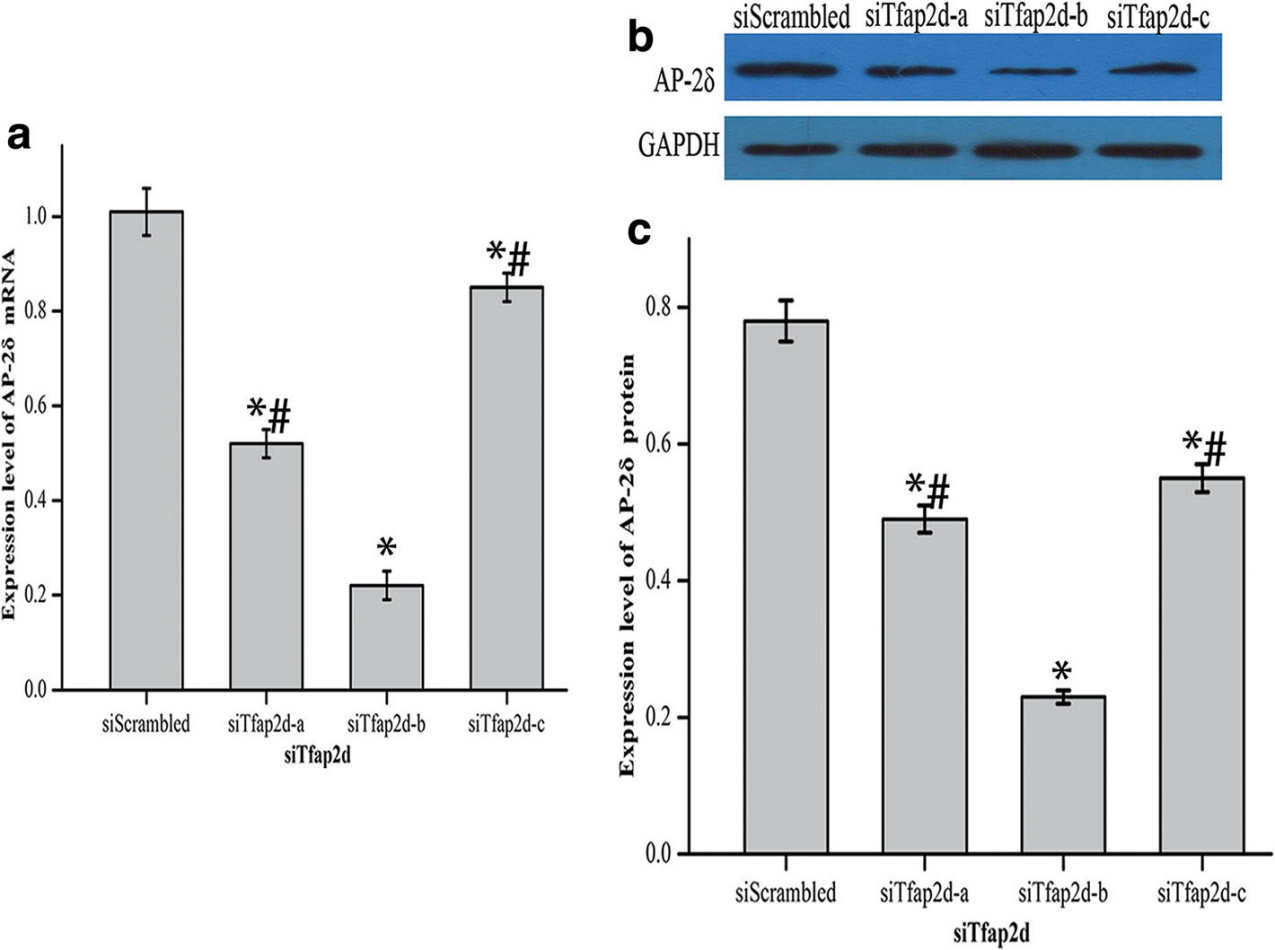
The siTfap2d-b fragment was characterized by the best specificity in knocking down AP-2δ. **a** HEI-OC1 cells were transfected with three siTfap2d fragments for 24 h and subjected to qRT-PCR to detect the expression level of AP-2δ mRNA. **b** Representative western blot of AP-2δ from cells treated with siTfap2d. GAPDH was used as an endogenous control. These images came from the same gel. **c** Expression level of AP-2δ protein in HEI-OC1 cells treated with siTfap2d. The data were normalized to GAPDH expression, and they were presented as the means±SD; n = 3 each group. * and # represent *P* < 0.05 compared with the group treated with the siScrambled fragment and the group treated with the siTfap2d-b fragment respectively

AP-2δ was preliminarily regarded as a TF that potentially regulated the expression of *Prestin*. To confirm whether TF AP-2δ binds to the transcriptional start site (TSS) of the Prestin gene promoter region, ChIP assay was carried out. An association between AP-2δ and S-1441 of the Prestin gene was observed in agarose gel electrophoresis after PCR, whereas there was no association with S-784. It demonstrated that AP-2δ was recruited to S-1441 of *Prestin*, as shown in Fig. 3a. The differences of enrichment of ChIP (S-1441 of *Prestin*) between the IP group and IgG group were statistically significant (Student’s *t*-test, *P*<0.05), it was shown in Fig. 4b, further suggesting that AP-2δ is involved in the transcriptional regulation of *Prestin*.

### AP-2δ had a negative regulatory role in Prestin expression

The ChIP experiment demonstrated that AP-2δ bound to *Prestin*. Next, small interfering RNA treatment was performed to explore the regulatory effect of AP-2δ on *Prestin*. Three AP-2δ siRNA fragments were designed and transfected into untreated HEI-OC1 cells, and qRT-PCR and western blot were used to screen the most effective siTfap2d fragment and to evaluate the function of AP-2δ. Figure 4 revealed that the expression of AP-2δ at mRNA and protein levels decreased obviously when cells transfected with siTfap2d-a, b, or c were compared with cells transfected with siScrambled (ANOVA, *P* < 0.05), and the differences between the group treated with siTfap2d-b and groups treated with siTfap2d-a, c were statistically significant (Tukey’s test, *P* < 0.05). This illustrated that the knockdown efficiency of siTfap2d-b was the highest.

**Fig. 5:**
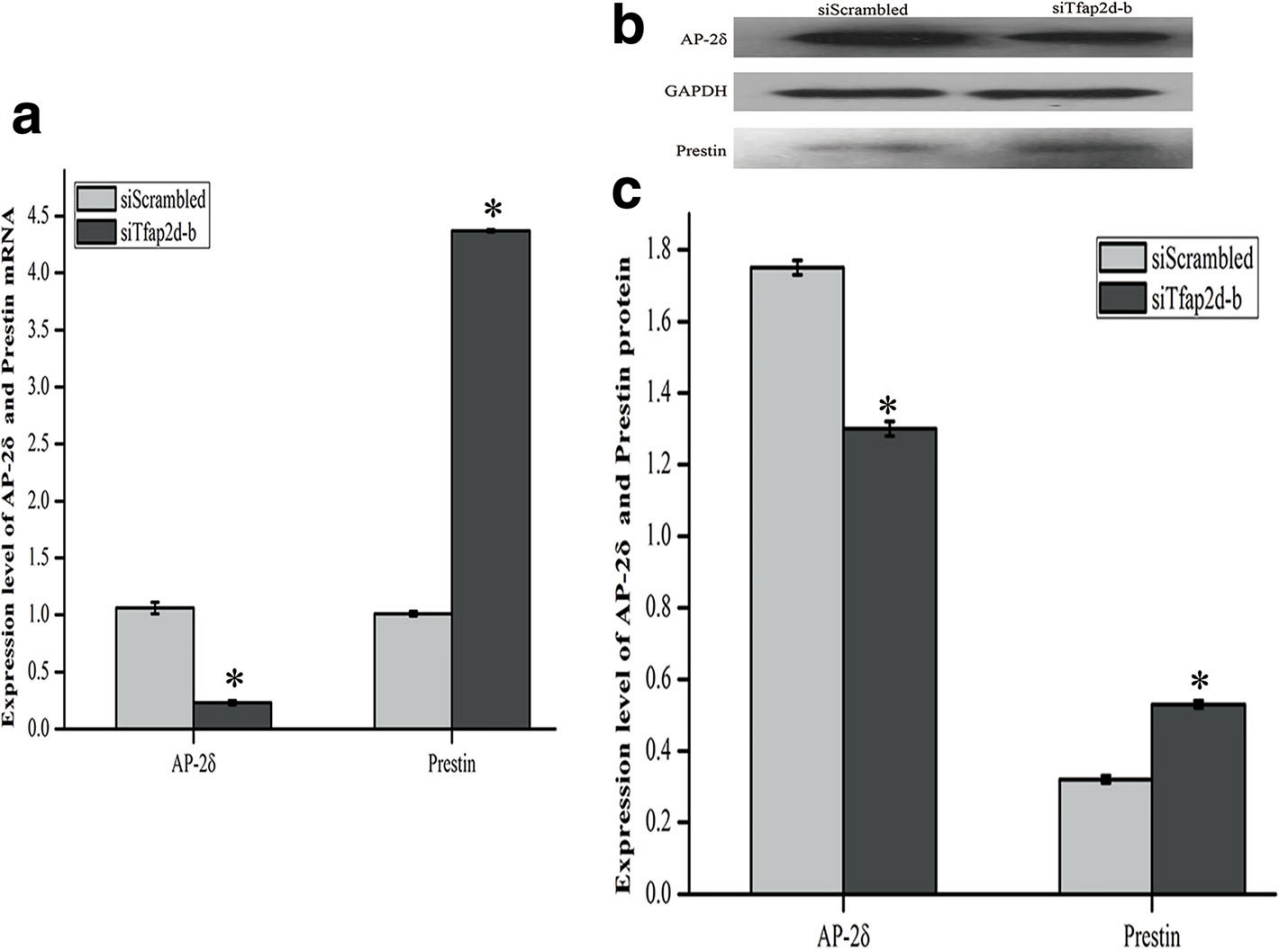
AP-2δ negatively regulated the transcription of Prestin. **a** HEI-OC1 cells were transfected with the siTfap2d-b fragment for 24 h and subjected to qRT-PCR to detect the expression level of AP-2δ and Prestin mRNA. **b** Representative western blot of AP-2δ and Prestin from cells treated with siTfap2d-b. GAPDH was used as an endogenous control. These images came from the same gel. **c** Expression level of AP-2δ and Prestin protein in HEI-OC1 cells treated with siTfap2d-b. The data were normalized to GAPDH expression, and they were presented as the means±SD; *n* = 3 each group. * represents *P* < 0.05 compared with the group treated with the siScrambled fragment

HEI-OC1 cells were transfected with siScrambled and siTfap2d-b fragment for 24 h and subjected to qRT-PCR and western blot to evaluate the effect of AP-2δ on *Prestin*. Figure 5 indicates that the expression of Prestin at mRNA and protein levels was elevated in HEI-OC1 cells with silenced AP-2δ, and there were significant differences between the groups (Student’s *t*-test, *P* < 0.05). It implied that AP-2δ may negatively regulate the transcription of *Prestin*.

### AP-2δ at mRNA and protein levels were down-regulated in HEI-OC1 cells upon oxidative stress

**Fig. 6:**
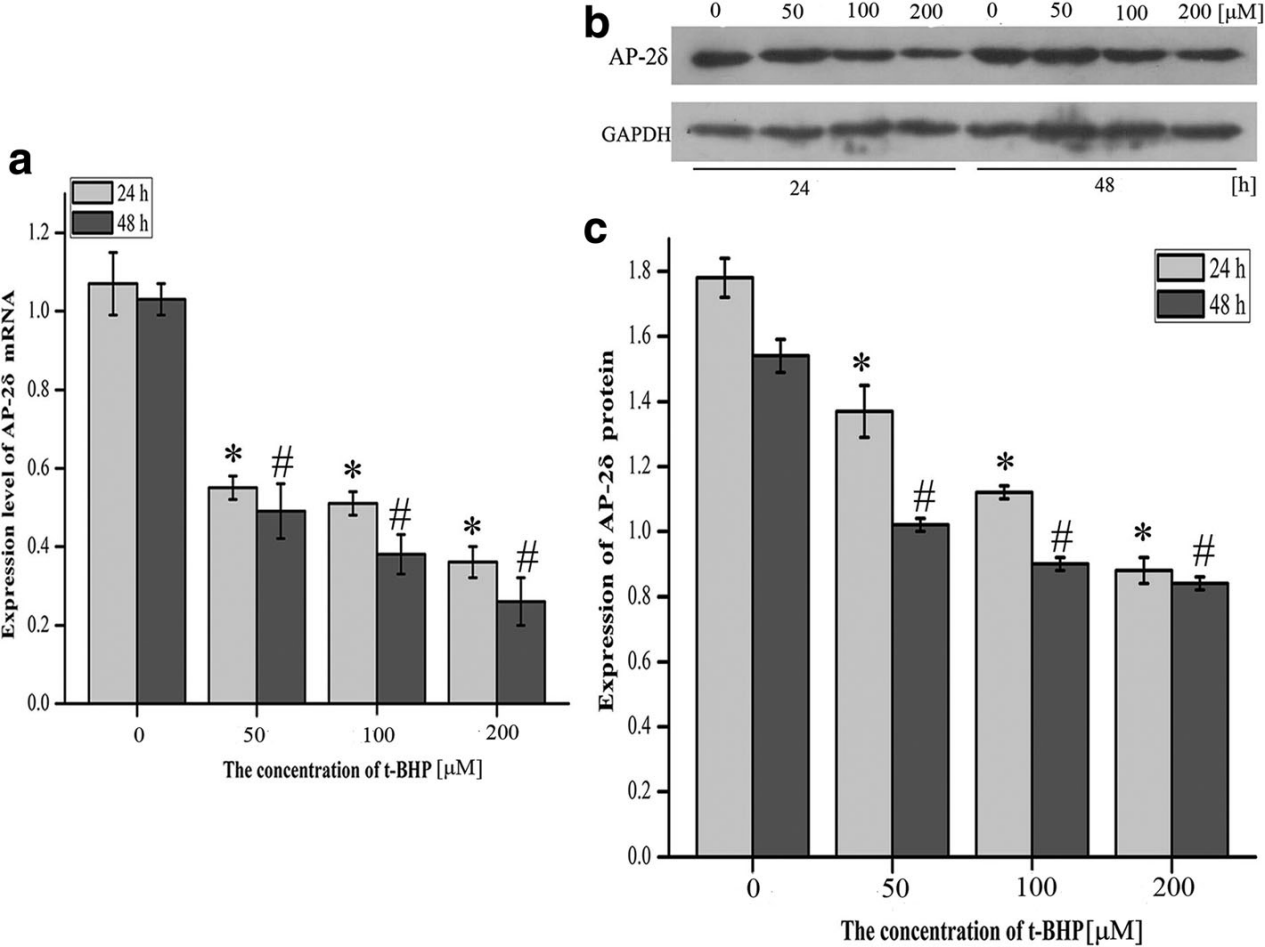
Expression level of AP-2δ in HEI-OC1 cells treated with t-BHP for 24 h and 48 h. **a** Expression level of AP-2δ mRNA in HEI-OC1 cells exposed to different concentrations of t-BHP (0 μM, 50 μM, 100 μM, 200 μM) for 24 h and 48 h. **b** Representative western blot of AP-2δ from cells treated with t-BHP. GAPDH was used as an endogenous control. These images came from the same gel. **c** Expression level of AP-2δ protein in HEI-OC1 cells exposed to t-BHP. The data were normalized to GAPDH expression, and they were presented as the means±SD; *n* = 3 each group. * and # represent *P* < 0.05 compared with the control group.

The expression level of AP-2δ is shown in Fig. 6. Oxidative stress induced decreased.

AP-2δ at the mRNA level and protein level in HEI-OC1 cells treated with t-BHP, and the higher the concentration of t-BHP was, the lower was the expression level of AP-2δ (Fig. 6a, c), and the differences between the experimental groups and the control group were significant (ANOVA and Tukey’s test, *P* < 0.05). Moreover, AP-2δ at both mRNA and protein levels after 48 h treatment was lower than after 24 h treatment.

## Discussion

The results revealed that oxidative stress induced a Prestin increase at the mRNA level but a concomitant decrease at the protein level. Also, the higher the concentration of t-BHP was, the higher was the Prestin mRNA level and the lower was the Prestin protein level. It illustrated that the expression of Prestin mRNA was activated and the expression of Prestin protein was suppressed when HEI-OC1 cells were treated with t-BHP, and the reaction was more apparent when damage to cells was more severe. Unfortunately, due to the existence of multiple oxidative stress regulatory systems in animal in vivo and the lack of a single research environment, it is difficult to verify the results of the cell model studies, which is also a limitation of the study. Combined with the above observations, it suggests that the protein level of Prestin is more sensitive to oxidative stress, and that a transcription mechanism is triggered to compensate the loss of the protein. When HEI-OC1 cells were treated with t-BHP, the level of ROS increased [22]. Proteins are among the main targets for oxidants due to their high rate constants for several reactions with ROS and their abundance in biological systems [30]. ROS can directly interact with proteins and cause their peroxidation and change their structure or function [31]. This might explain why the Prestin protein level decreased. The formation of protein requires the participation of transcription, post-transcriptional regulation and translation regulation, and it takes a period of time for Prestin mRNA to be translated and processed into Prestin protein, which explains why the Prestin protein level was higher after 48 h exposure than that after 24 h exposure. Thus it can be speculated that the regulation of Prestin in HEI-OC1 cells upon oxidative stress occurs at the transcriptional level.

Based on the above hypothesis, we searched for TFs that could modulate the Prestin gene. There were 8 TFs found: AP-2α, AP-2β, AP-2γ, AP-2δ, AP-2ε, COE3, TBXA5 and TBX22. The expression of AP-2δ mRNA under oxidative stress showed the strongest correlation. It indicated that AP-2δ specifically bound to the Prestin gene and negatively regulated its expression based on the results of siRNA and ChIP experiments. Interestingly, oxidative stress induced decrease of AP-2δ at the mRNA level and protein level in HEI-OC1 cells treated with t-BHP, and the higher the concentration of t-BHP was and the longer the exposed time, the lower was the expression level of AP-2δ, while oxidative stress induced an increase of Prestin mRNA, which revealed that AP-2δ suppression further boosted Prestin mRNA activation.

TF AP-2δ is one of the five subtypes (AP-2α, AP-2β, AP-2γ, AP-2δ, and AP-2ε) of AP-2 family members in mammals. All mammalian AP-2 proteins, except AP-2δ, share a common highly conserved sequence and structure, and they play an important role in cell proliferation, differentiation, apoptosis, and carcinogenesis by regulating the transcription of target genes through binding to a specific sequence [32]. AP-2δ is a divergent member among AP-2 proteins because of the absence of the PY motif. It is encoded by the *Tfap2d* gene, and its expression is mainly restricted to the developing heart, central nervous system and retina in mice [33, 34]. Li et al. [33] found that auditory information could still be recorded in AP-2δ-deficient mice that lack a central part of the auditory pathway. They also showed that *Brn3c* (*Pou4f3*) was a gene targeted and positively regulated by AP-2δ. *Brn3c* (*Pou4f3*) plays an essential role in cell maturation and the survival of cochlea hair cells in inner ear sensory epithelia [35], and it participates in the modulation of the Prestin gene [18]. To maintain homeostasis and to protect cells, mitochondria produce not only ROS but also superoxide dismutase 2 (SOD2) under oxidative stress conditions [36]. SOD2 single nucleotide polymorphisms (SNPs) are associated with age-related deafness or noise-induced deafness [37], and mutations in different promoter regions lead to different binding affinities to AP2 proteins, thus regulating AP2 protein and influencing hearing. Nolan et al. [38] made the same observation in inner-ear-derived cell lines and populations in London. The above studies indicate that AP-2δ protein plays a pivotal role in the development and function of the hearing apparatus, although there has so far been no evidence that *Prestin* is a target of AP-2δ. In this study, ChIP assay confirmed that AP-2δ occupied the Prestin promoter in HEI-OC1 cells. However, genes and proteins are characterized by spatial and temporal expression patterns in each stage of development in the inner ear of animals and humans; therefore, it is necessary to carry out relevant experimental verification at the animal or the human level. In addition, a large number of downstream target genes are modulated by the same TF due to the limited total TF pool, and downstream gene function has both specificity and crosstalk. Therefore, it is vital to further explore the upstream and downstream factors related to AP-2δ involvement in the transcription of *Prestin* in order to elucidate the overall signaling pathway, which will provide a detailed understanding of the molecular mechanisms and a theoretical basis for the biological treatment of sensory deafness. This research revealed that decrease of AP-2δ expression level in HEI-OC1 under oxidative stress conditions perhaps aggravated the increase of Prestin at both the mRNA level and the protein level, reflecting a compensatory mechanism to maintain the expression of Prestin protein in cells.

## Conclusions

This study revealed that oxidative stress induced Prestin increase at the mRNA level but with a concomitant decrease at the protein level. Also, the expression of Prestin at both the mRNA level and the protein level in HEI-OC1 cells cultured with t-BHP for 48 h was higher than that for 24 h in experimental groups, which indicated a compensatory repair mechanism. AP-2δ is one of the important TFs modulating the Prestin gene, and plays a negative regulatory role at the transcriptional level. Under the oxidative stress state, AP-2δ was down-regulated to aggravate the increase of Prestin to maintain the expression of Prestin protein in cells. However, the regulatory pathway of AP-2δ is still unclear, and searching for other factors regulated by AP-2δ is our next goal. In addition, the seven remaining potential TFs of the Prestin gene should be further verified.

## Additional file

Additional file 1: **Figure S1.** The schematic diagram of mice the prestin gene showing the targeted positions of probes sequences. **Table S1.** The information of potential proteins binding to the Prestin gene. (DOC 425 kb)

## Data Availability

All data generated or analyzed during this study are included in this. article and its Additional file 1.

## Abbreviations

AP-2δ: Activating enhancer binding protein-2δ
HEI-OC1 cells: House Ear Institute-Organ of Corti 1 cells
OHCs: Outer hair cells
qRT-PCR: Quantitative real-time polymerase chain reaction
reverse ChIP: Reverse chromatin immunoprecipitation
ROS: Reactive oxygen species
siRNA: Small interfering RNA
t-BHP: Tert-butyl hydroperoxide
TF: Transcription factor
TSS: Transcriptional start site
