# Supplementary material for: The effect of AP-2δ on transcription of the Prestin gene in HEI-OC1 cells upon oxidative stress
Source: Cell Mol Biol Lett. 2019 Jun 26;24:45. doi: 10.1186/s11658-019-0170-0 (PMC6595603; doi:10.1186/s11658-019-0170-0)
Supplement: Supplementary file 1 — Figure S1. The schematic diagram of mice the prestin gene showing the targeted positions of probes sequences. Table S1. The information of potential proteins binding to the Prestin gene. (DOC 425 kb) [file 11658_2019_170_MOESM1_ESM.doc]

**The effect of AP-2δ on transcription of the Prestin gene in HEI-OC1 cells upon oxidative stress**

Xuan Luo, Yun Xia, Xu-Dong Li, Jun-Yi Wang^*^


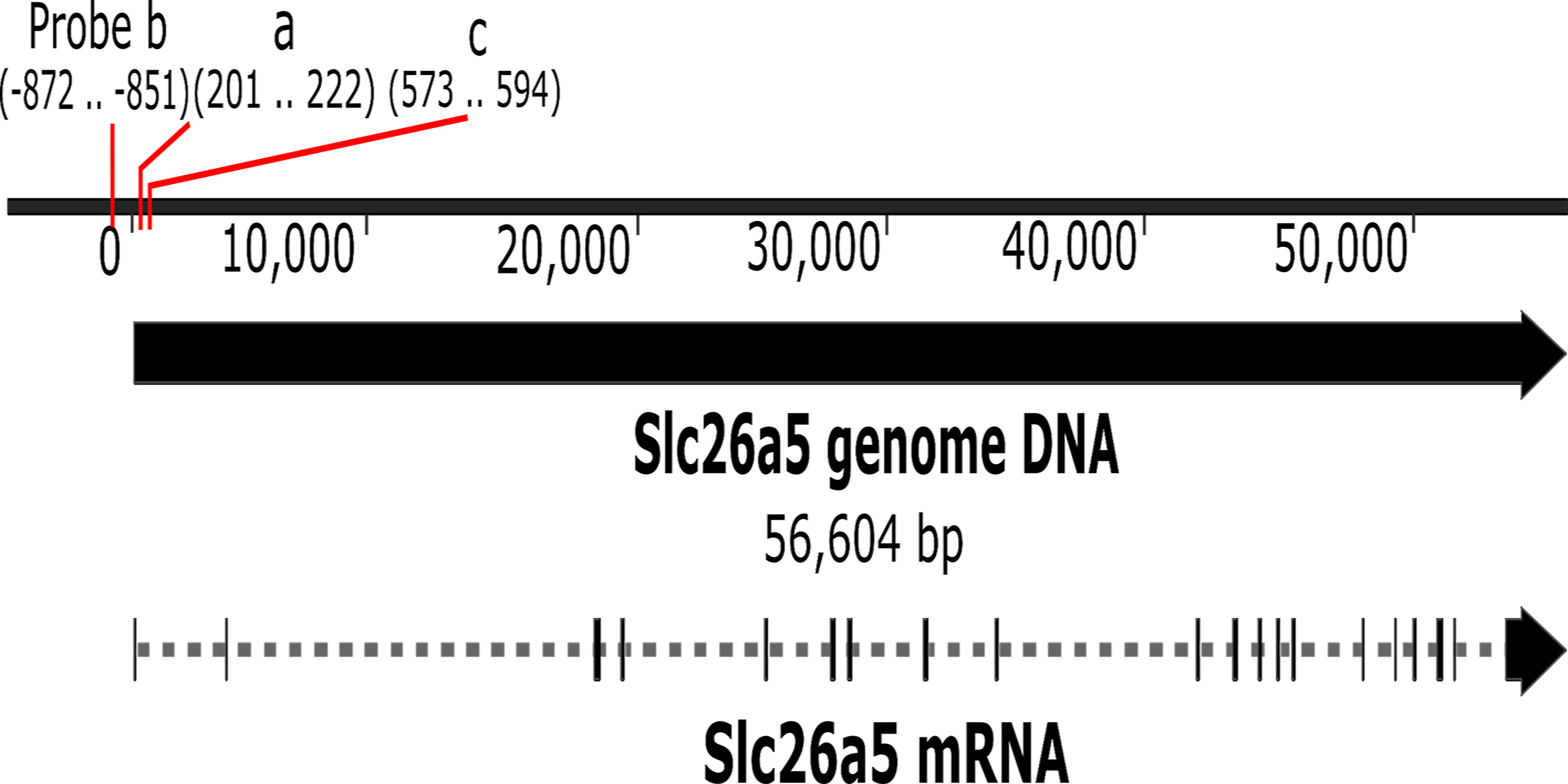


**Fig. S1** The schematic diagram of mice the Prestin gene showing the targeted positions of probes sequences.

Slc26a5 is the alias of *Prestin*, Prestin probes used in reverse Chip assay are targeted to the promoter region of *Prestin*.

### Table S1 The information of potential proteins binding to the Prestin gene

| Abbreviation | Full name | Gene |
| --- | --- | --- |
| K1C10 | Keratin, type I cytoskeletal 10 | *Krt10* |
| 2A5A | Serine/threonine-protein phosphatase 2A 56 kDa regulatory subunit alpha isoform | *Ppp2r5a* |
| ACTB | Actin, cytoplasmic 1 | *Actb* |
| ACTBL | Beta-actin-like protein 2 | *Actbl2* |
| ACTS | Actin, alpha skeletal muscle | *Acta1* |
| ACVR1 | Activin receptor type-1 | *Acvr1* |
| ACYP1 | Acylphosphatase-1 | *Acyp1* |
| AKT1 | RAC-alpha serine/threonine-protein kinase | *Akt1* |
| AP-2α | Transcription factor AP-2-alpha | *Tfap2a* |
| AP-2β | Transcription factor AP-2-beta | *Tfap2b* |
| AP-2γ | Transcription factor AP-2 gamma | *Tfap2c* |
| AP-2δ | Transcription factor AP-2-delta | *Tfap2d* |
| AP-2ε | Transcription factor AP-2-epsilon | *Tfap2e* |
| APC | Adenomatous polyposis coli protein | *Apc* |
| ARHG1 | Rho guanine nucleotide exchange factor 1 | *Arhgef1* |
| ARHG6 | Rho guanine nucleotide exchange factor 6 | *Arhgef6* |
| ATP5J | ATP synthase-coupling factor 6, mitochondrial | *Atp5j* |
| ATPA | ATP synthase subunit alpha, mitochondrial | *Atp5a1* |
| ATPB | ATP synthase subunit beta, mitochondrial | *Atp5b* |
| BAZ1B | Tyrosine-protein kinase BAZ1B | *Baz1b* |
| BRD8 | Bromodomain-containing protein 8 | *Brd8* |
| CAC1A | Voltage-dependent P/Q-type calcium channel subunit alpha-1A | *Cacna1a* |
| CAC1E | Voltage-dependent R-type calcium channel subunit alpha-1E | *Cacna1e* |
| CCAR1 | Cell division cycle and apoptosis regulator protein 1 | *Ccar1* |
| CHD7 | Chromodomain-helicase-DNA-binding protein 7 | *Chd7* |
| CLIP1 | CAP-Gly domain-containing linker protein 1 | *Clip1* |
| CO7A1 | Collagen alpha-1(VII) chain | *Col7a1* |
| CO8B | Complement component C8 beta chain | *C8b* |
| COE3 | Transcription factor COE3 | *Ebf3* |
| COQ6 | Ubiquinone biosynthesis monooxygenase COQ6 | *Coq6* |
| CP7A1 | Cholesterol 7-alpha-monooxygenase | *Cyp7a1* |
| CPNE3 | Copine-3 | *Cpne3* |
| CSRP3 | Cysteine and glycine-rich protein 3 | *Csrp3* |
| CTP5A | Contactin-associated protein like 5-1 | *Cntnap5a* |
| CTP5B | Contactin-associated protein like 5-2 | *Cntnap5b* |
| DPOLZ | DNA polymerase zeta catalytic subunit | *Rev3l* |
| EF1A2 | Elongation factor 1-alpha 2 | *Eef1a2* |
| EMID1 | EMI domain-containing protein 1 | *Emid1* |
| F158A | UPF0172 protein FAM158A | *Fam158a* |
| FAM21 | WASH complex subunit FAM21 | *Fam21* |
| FKB1A | Peptidyl-prolyl cis-trans isomerase FKBP1A | *Fkbp1a* |
| FLNA | Filamin-A | *Flna* |
| GDPD1 | Glycerophosphodiester phosphodiesterase domain-containing protein 1 | *Gdpd1* |
| GFAP | Glial fibrillary acidic protein | *Gfap* |
| GLT13 | Polypeptide N-acetylgalactosaminyltransferase 13 | *Galnt13* |
| GOGA5 | Golgin subfamily A member 5 | *Golga5* |
| GON4L | GON-4-like protein | *Gon4l* |
| GP160 | Probable G-protein coupled receptor 160 | *Gpr160* |
| GRAM3 | GRAM domain-containing protein 3 | *Gramd3* |
| H12 | Histone H1.2 | *Hist1h1c* |
| H13 | Histone H1.3 | *Hist1h1d* |
| H14 | Histone H1.4 | *Hist1h1e* |
| H1T | Histone H1t | *Hist1h1t* |
| H2A1F | Histone H2A type 1-F | *Hist1h2af* |
| H2AV | Histone H2A.V | *H2afv* |
| H2AX | Histone H2A.x | *H2afx* |
| H2B1A | Histone H2B type 1-A | *Hist1h2ba* |
| H2B1B | Histone H2B type 1-B | *Hist1h2bb* |
| H2B1C | Histone H2B type 1-C/E/G | *Hist1h2bc* |
| H2B2E | Histone H2B type 2-E | *Hist2h2be* |
| H4 | Histone H4 | *Hist1h4a* |
| HBA | Hemoglobin subunit alpha | *Hba* |
| HBAZ | Hemoglobin subunit zeta | *Hbz* |
| HBB1 | Hemoglobin subunit beta-1 | *Hbb-b1* |
| HNRPK | Heterogeneous nuclear ribonucleoprotein K | *Hnrnpk* |
| HPCL1 | Hippocalcin-like protein 1 | *Hpcal1* |
| HUWE1 | E3 ubiquitin-protein ligase HUWE1 | *Huwe1* |
| K1C13 | Keratin, type I cytoskeletal 13 | *Krt13* |
| K1C14 | Keratin, type I cytoskeletal 14 | *Krt14* |
| K1C15 | Keratin, type I cytoskeletal 15 | *Krt15* |
| K1C16 | Keratin, type I cytoskeletal 16 | *Krt16* |
| K1C17 | Keratin, type I cytoskeletal 17 | *Krt17* |
| K1C18 | Keratin, type I cytoskeletal 18 | *Krt18* |
| K1C19 | Keratin, type I cytoskeletal 19 | *Krt19* |
| K1C20 | Keratin, type I cytoskeletal 20 | *Krt20* |
| K1C24 | Keratin, type I cytoskeletal 24 | *Krt24* |
| K1C28 | Keratin, type I cytoskeletal 28 | *Krt28* |
| K1C40 | Keratin, type I cytoskeletal 40 | *Krt40* |
| K1C42 | Keratin, type I cytoskeletal 42 | *Krt42* |
| K1H1 | Keratin, type I cuticular Ha1 | *Krt31* |
| K1H2 | Keratin, type I cuticular Ha2 | *Krt32* |
| K22E | Keratin, type II cytoskeletal 2 epidermal | *Krt2* |
| K22O | Keratin, type II cytoskeletal 2 oral | *Krt76* |
| K2C1 | Keratin, type II cytoskeletal 1 | *Krt1* |
| K2C1B | Keratin, type II cytoskeletal 1b | *Krt77* |
| K2C4 | Keratin, type II cytoskeletal 4 | *Krt4* |
| K2C5 | Keratin, type II cytoskeletal 5 | *Krt5* |
| K2C6A | Keratin, type II cytoskeletal 6A | *Krt6a* |
| K2C6B | Keratin, type II cytoskeletal 6B | *Krt6b* |
| K2C7 | Keratin, type II cytoskeletal 7 | *Krt7* |
| K2C71 | Keratin, type II cytoskeletal 71 | *Krt71* |
| K2C72 | Keratin, type II cytoskeletal 72 | *Krt72* |
| K2C73 | Keratin, type II cytoskeletal 73 | *Krt73* |
| K2C74 | Keratin, type II cytoskeletal 74 | *Krt74* |
| K2C75 | Keratin, type II cytoskeletal 75 | *Krt75* |
| K2C79 | Keratin, type II cytoskeletal 79 | *Krt79* |
| K2C8 | Keratin, type II cytoskeletal 8 | *Krt8* |
| KAT5 | Histone acetyltransferase KAT5 | *Kat5* |
| KLBL4 | Plasma kallikrein-like protein 4 | *Klkbl4* |
| KPCE | Protein kinase C epsilon type | *Prkce* |
| KR168 | Keratin-associated protein 16-8 | *Krtap16-8* |
| KRT35 | Keratin, type I cuticular Ha5 | *Krt35* |
| KRT36 | Keratin, type I cuticular Ha6 | *Krt36* |
| KRT84 | Keratin, type II cuticular Hb4 | *Krt84* |
| KRT85 | Keratin, type II cuticular Hb5 | *Krt85* |
| KS6A4 | Ribosomal protein S6 kinase alpha-4 | *Rps6ka4* |
| KS6A5 | Ribosomal protein S6 kinase alpha-5 | *Rps6ka5* |
| KT222 | Keratin-like protein KRT222 | *Krt222* |
| KT33B | Keratin, type I cuticular Ha3-II | *Krt33b* |
| LAMB2 | Laminin subunit beta-2 | *Lamb2* |
| LAMC1 | Laminin subunit gamma-1 | *Lamc1* |
| LIMC1 | LIM and calponin homology domains-containing protein 1 | *Limch1* |
| LRRC6 | Leucine-rich repeat-containing protein 6 | *Lrrc6* |
| LT4R1 | Leukotriene B4 receptor 1 | *Ltb4r* |
| LZTL1 | Leucine zipper transcription factor-like protein 1 | *Lztfl1* |
| MA2B1 | Lysosomal alpha-mannosidase | *Man2b1* |
| MAP1B | Microtubule-associated protein 1B | *Map1b* |
| MAP1S | Microtubule-associated protein 1S | *Map1s* |
| MCCA | Methylcrotonoyl-CoA carboxylase subunit alpha, mitochondrial | *Mccc1* |
| MDM20 | N-terminal acetyltransferase B complex subunit MDM20 | *Mdm20* |
| METL2 | Methyltransferase-like protein 2 | *Mettl2* |
| MTA70 | N6-adenosine-methyltransferase 70 kDa subunit | *Mettl3* |
| MY18A | Myosin-XVIIIa | *Myo18a* |
| MYH10 | Myosin-10 | *Myh10* |
| MYH9 | Myosin-9 | *Myh9* |
| MYL4 | Myosin light chain 4 | *Myl4* |
| NAL4B | NACHT, LRR and PYD domains-containing protein 4B | *Nlrp4b* |
| NCOA5 | Nuclear receptor coactivator 5 | *Ncoa5* |
| NPAT | Protein NPAT | *Npat* |
| NPTXR | Neuronal pentraxin receptor | *Nptxr* |
| NR4A2 | Nuclear receptor subfamily 4 group A member 2 | *Nr4a2* |
| NR4A3 | Nuclear receptor subfamily 4 group A member 3 | *Nr4a3* |
| NT5D1 | 5'-nucleotidase domain-containing protein 1 | *Nt5dc1* |
| NUDC2 | NudC domain-containing protein 2 | *Nudcd2* |
| NUP53 | Nucleoporin NUP53 | *Nup35* |
| OGR1 | Ovarian cancer G-protein coupled receptor 1 | *Gpr68* |
| PARP1 | Poly [ADP-ribose] polymerase 1 | *Parp1* |
| PCATB | Lysophosphatidylcholine acyltransferase 2-B | *Aytl1b* |
| PCLO | Protein piccolo | *Pclo* |
| PERE | Eosinophil peroxidase | *Epx* |
| PHF23 | PHD finger protein 23 | *Phf23* |
| PHF5A | PHD finger-like domain-containing protein 5A | *Phf5a* |
| PIWL4 | Piwi-like protein 4 | *Piwil4* |
| PLA1A | Phospholipase A1 member A | *Pla1a* |
| PLCD | 1-acyl-sn-glycerol-3-phosphate acyltransferase delta | *Agpat4* |
| PRCM | Probable proline racemase | *--* |
| RHGBA | Rho GTPase-activating protein 11A | *Arhgap11a* |
| RL37A | 60S ribosomal protein L37a | *Rpl37a* |
| RP1 | Oxygen-regulated protein 1 | *Rp1* |
| RP1L1 | Retinitis pigmentosa 1-like 1 protein | *Rp1l1* |
| RPOM | DNA-directed RNA polymerase, mitochondrial | *Polrmt* |
| RUFY3 | Protein RUFY3 | *Rufy3* |
| SBP2L | Selenocysteine insertion sequence-binding protein 2-like | *Secisbp2l* |
| SEM4D | Semaphorin-4D | *Sema4d* |
| SEPT1 | Septin-1 | *Sept1* |
| SFRS1 | Splicing factor, arginine/serine-rich 1 | *Sfrs1* |
| SNIP1 | Smad nuclear-interacting protein 1 | *Snip1* |
| SSDH | Succinate-semialdehyde dehydrogenase, mitochondrial | *Aldh5a1* |
| SYMPK | Symplekin | *Sympk* |
| SYUA | Alpha-synuclein | *Snca* |
| TBX22 | T-box transcription factor TBX22 | *Tbx22* |
| TBX5 | T-box transcription factor TBX5 | *Tbx5* |
| TCAM1 | TIR domain-containing adapter molecule 1 | *Ticam1* |
| TCP4 | Activated RNA polymerase II transcriptional coactivator p15 | *Sub1* |
| TCPR1 | Tectonin beta-propeller repeat-containing protein 1 | *Tecpr1* |
| TDH | L-threonine 3-dehydrogenase, mitochondrial | *Tdh* |
| TEP1 | Telomerase protein component 1 | *Tep1* |
| TES | Testin | *Tes* |
| THIM | 3-ketoacyl-CoA thiolase, mitochondrial | *Acaa2* |
| TIRAP | Toll/interleukin-1 receptor domain-containing adapter protein | *Tirap* |
| TP8L3 | Tumor necrosis factor alpha-induced protein 8-like protein 3 GN= PE=2 SV=1 | *Tnfaip8l3* |
| TPD52 | Tumor protein D52 GN= PE=1 SV=2 | *Tpd52* |
| TRPV3 | Transient receptor potential cation channel subfamily V member 3 GN= PE=2 SV=2 | *Trpv3* |
| TXLNG | Gamma-taxilin GN= PE=2 SV=1 | *Txlng* |
| UBIQ | Ubiquitin GN= PE=1 SV=1 | *Rps27a* |
| UBR4 | E3 ubiquitin-protein ligase UBR4 | *Ubr4* |
| UN13D | Protein unc-13 homolog D | *Unc13d* |
| VP13B | Vacuolar protein sorting-associated protein 13B | *Vps13b* |
| WDR67 | WD repeat-containing protein 67 | *Wdr67* |
| YE019 | Probable ubiquitin-conjugating enzyme E2 FLJ25076 homolog | *--* |
| YTDC2 | Probable ATP-dependent RNA helicase YTHDC2 | *Ythdc2* |
| ZN318 | Zinc finger protein 318 (Fragment) | *Znf318* |
| ZN598 | Zinc finger protein 598 | *Znf598* |
